# Adverse Drug Reaction Prediction Using Scores Produced by Large-Scale Drug-Protein Target Docking on High-Performance Computing Machines

**DOI:** 10.1371/journal.pone.0106298

**Published:** 2014-09-05

**Authors:** Montiago X. LaBute, Xiaohua Zhang, Jason Lenderman, Brian J. Bennion, Sergio E. Wong, Felice C. Lightstone

**Affiliations:** 1 Computational Engineering Division, Lawrence Livermore National Laboratory, Livermore, California, United States of America; 2 Biosciences and Biotechnology Division, Lawrence Livermore National Laboratory, Livermore, California, United States of America; Kyushu University, Japan

## Abstract

Late-stage or post-market identification of adverse drug reactions (ADRs) is a significant public health issue and a source of major economic liability for drug development. Thus, reliable *in silico* screening of drug candidates for possible ADRs would be advantageous. In this work, we introduce a computational approach that predicts ADRs by combining the results of molecular docking and leverages known ADR information from DrugBank and SIDER. We employed a recently parallelized version of AutoDock Vina (VinaLC) to dock 906 small molecule drugs to a virtual panel of 409 DrugBank protein targets. L1-regularized logistic regression models were trained on the resulting docking scores of a 560 compound subset from the initial 906 compounds to predict 85 side effects, grouped into 10 ADR phenotype groups. Only 21% (87 out of 409) of the drug-protein binding features involve known targets of the drug subset, providing a significant probe of off-target effects. As a control, associations of this drug subset with the 555 annotated targets of these compounds, as reported in DrugBank, were used as features to train a separate group of models. The Vina off-target models and the DrugBank on-target models yielded comparable median area-under-the-receiver-operating-characteristic-curves (AUCs) during 10-fold cross-validation (0.60–0.69 and 0.61–0.74, respectively). Evidence was found in the PubMed literature to support several putative ADR-protein associations identified by our analysis. Among them, several associations between neoplasm-related ADRs and known tumor suppressor and tumor invasiveness marker proteins were found. A dual role for interstitial collagenase in both neoplasms and aneurysm formation was also identified. These associations all involve off-target proteins and could not have been found using available drug/on-target interaction data. This study illustrates a path forward to comprehensive ADR virtual screening that can potentially scale with increasing number of CPUs to tens of thousands of protein targets and millions of potential drug candidates.

## Introduction

Adverse drug reactions (ADRs) are detrimental, rare and complex perturbations of biological pathways by pharmacologically active small molecules. Each year ADRs cause 100,000 fatalities in the US [1]. One cost estimate of drug-related morbidity and mortality is $177 billion annually [2], which is comparable to the public health burden of chronic illnesses like diabetes ($245 billion in 2012 [3]). A systematic and accurate capability for reliably ruling out severe ADRs early in the drug development process currently does not exist. As a result, billions of research and development dollars are wasted as drugs present with serious ADRs either in late stage development or post-market approval. Highly publicized examples of phase IV failures include rosiglitazone (“Avandia”) [4] and rofecoxib (“Vioxx”) [5]. Early identification of serious ADRs would be ideal.

Although many ADRs are multi-factorial and depend on patient- and treatment-specific factors (*e.g.* genetic polymorphisms and medical history of the patient, treatment dosages, environmental exposures, dynamics and kinetics of the relevant systems biology, etc.), all ADRs are initiated by the binding of a drug molecule to a target, whether these binding events are intended, on-target binding or promiscuous binding to one or more off-target proteins. Currently, pharmaceutical companies commonly employ experimental *in vitro* toxicity panels to assay small molecule binding to potentially critical protein receptors [6]. Unfortunately, these panels probably do not include all of the proteins and receptors needed for high-accuracy prediction of serious ADRs [7]. Even if it were known how to augment toxicity panels to include a minimally complete set of receptors relevant for serious ADRs, there is uncertainty about how efficiently it could be screened.

An *in silico* platform that could accurately predict serious ADRs prior to costly *in vitro* screening panels and clinical safety trials is highly desirable and has been the focus of several recent studies.

A popular approach is to data-mine the publicly available databases for experimentally elucidated interrelationships between the chemical structures of drugs, their known interactions with proteins (most often their intended targets), and their known ADR profiles. An early study by Fliri and co-workers [8] clustered drugs based on their ability to inhibit a selected set of proteins. They showed that similar inhibition profiles indicate a similar set of side effects. More recently, Cobanoglu and co-workers [9] performed probabilistic matrix factorization on a 1,413 drug×1,050 known target protein matrix to learn a latent variable correlation structure between drugs and proteins. Drugs were then clustered in this latent variable space, and it was found that drugs with similar therapeutic actions clustered together, independent of similarities in chemical structure. A highly cited effort by Campillos *et al.* [10] indicated that drugs with similar side effects have a correspondingly similar profile of protein targets. Another series of studies applied statistical machine learning approaches like support vector machines and sparse canonical correlation analysis (SCCA) to publicly available datasets to train models for ADR prediction. Pauwels *et al.* [11] used SCCA to relate PubChem [12] chemical substructure fingerprints of 888 approved drugs to 1385 side effects in SIDER. Yamanishi and co-workers [13] used a similar approach to integrate drug-protein target data found in DrugBank and Matador with PubChem fingerprints to predict 969 SIDER side effects, applying both SCCA and a kernel regression method. They used the models to predict side effects in 730 previously uncharacterized small molecules in DrugBank, where side-effect information was not available in SIDER. Finally, Liu *et al.* [14] found that adding phenotypic data on the drug (*i.e.* the presence or absence of side effects, excluding the one being predicted) to a similar feature representation to that considered in [13] greatly enhances prediction of the ADR of interest, obtaining AUCs>0.9. However, since their approach relies on health outcomes data on the drug compound, the method is unsuitable for ADR prediction in the early-stage development of nascent drug compounds, prior to *in vitro* studies or clinical trials. In all of the cases listed above, only global quality-of-performance metrics, aggregated across all considered side effects, are reported, making it difficult to assess how the models performed on individual side effects or classes of side effects.

There is another group of studies that more fully exploit the network structure of drug, protein, and ADR entity relationships. A network-oriented approach by Cami [15] analyzed a dataset consisting of 809 drug feature vectors (consisting of drug features from DrugBank and PubChem) and proprietary data on the drug side effect profiles. A unique aspect of the dataset is that the time ordering of when specific side effects appeared is reported. Starting with side effect profiles on the drugs from 2005, they trained a logistic regression model that could predict the side effects that manifested between 2006–2010, preserving the temporal order of how they manifest. The preservation of the time-ordering of the side effect appearance is appealing, but it is unclear how their approach would generalize to a different dataset. Mizutani [16] applied SCCA to find relationships between the drug-protein interaction network of 658 drugs from DrugBank and 1368 proteins extracted from DrugBank and Matador [17] databases to 1339 side effects associations as found in SIDER [18]. They found significant enrichment in most of the correlated protein-side effect sets for proteins involved in the same KEGG [19] and Gene Ontology biological pathways [20]. Similarly, Kuhn [21] constructed an explicit network to predict and characterize proteins that cause side effects by drawing statistical inferences between drug-target and drug-ADR links. Their method is able to reveal causal relationships between targets and ADRs but is highly sensitive to outliers. For instance, there was insufficient statistical power to associate side effects to proteins that were an off-target of only a small number of drugs.

Indeed, the main weakness of these QSAR-like studies is their reliance on what is present in experimental data, which will tend to feature a strong bias towards approved drugs (*i.e.* little representation of serious ADRs) and on-target or intended effects. It is difficult to see how analysis of drug-intended target binding data could be applied to explore correlations between off-target drug-protein binding and possibly rare ADRs.

Recently, systems biology approaches have been used to predict ADRs by viewing ADRs as perturbations of biological pathways. These approaches seek to transcend the “one drug-one target” paradigm used in traditional drug design, which ignores system-wide effects that cause a drug to have unforeseen pharmacological effects [22]. Scheiber *et al.* [23] integrated several chemical and biological databases by comparing perturbed and unperturbed pathways in a set of compounds that have a common toxicity phenotype. They use this analysis to link pathways with particular ADRs. Huang and co-workers [24] combined clinical observation data with drug-target data and the gene ontology (GO) annotations of the target proteins to predict ADRs. They find a significant improvement in the quality of their models by incorporating features from the protein-protein interaction (PPI) network of the targets. Similarly, Huang *et al.* [25] increased the median AUCs of their support vector machine models from 0.591 to 0.700 by adding both PPI network and small molecule structural features to their feature set.

In all of these cited cases, the efforts to solve the ADR prediction problem have focused on integrating publicly available and (in some cases proprietary) biological (*e.g.* physical and chemical small molecule properties, drug-protein associations, protein-protein interaction networks, biological pathway and gene annotations, etc.) and epidemiological data on side effect-related health outcomes (*e.g.* FDA package label data, clinical trial data) to train statistical models to predict ADRs with various degrees for success.

A key drawback of using experimental data is that the type and quality of data that exists is influenced as much by the financial limitations of experimental drug development as by the relevant biological science. The drug-protein associations aggregated from DrugBank and Matador can be represented as a Boolean matrix where ‘1’s (‘0’s) would indicate the presence (absence) of an association. This matrix has been used for some of the previous efforts, as noted above, and is highly sparse with ‘0’s indicating both negative results of assays and unperformed assays. ADR-protein associations derived from these data limit us to patterns in known, intended “on-target” associations and limit the ability to find novel off-target associations. Also, data on lead compounds that have failed in the development pipeline are typically regarded as proprietary information and are generally unavailable for inclusion in analysis. Clearly, the majority of publicly available data is biased in ways which are difficult to correct.

An alternative approach is to leverage ever-growing databases of high-resolution, experimentally-solved, protein structures, such as the Protein Data Bank (PDB) [26], and use molecular modeling to infer putative off-target interactions of drugs with known ADRs. Technical advances in drug-protein binding modeling, protein sequencing, and homology modeling allow high-throughput virtual screening early in the drug discovery process. Vast libraries of small molecules can be docked to a large array of protein structures in order to simultaneously predict putative drug targets and ancillary off-target binding interactions that may have associations to serious ADRs. Yang *et al.* [27] used virtual docking to propose possible interactions between a set of 845 proteins and a set of 162 drugs that induced at least one of four ADRs. Lounkine *et al.* [28] predicted the activity of 656 marketed drugs on 73 targets from the Novartis *in vitro* safety panel using the similarity ensemble approach (SEA). This was not a true docking study *per se*, in that SEA calculates the chemical similarity of each drug with each of the native ligands of the 73 targets.

Two previous efforts, in particular, are similar to our current study. First, Wallach and co-workers [29] applied multiple stages of logistic regression to docking scores involving 730 drugs, 830 human protein targets and then applied multiple stages of logistic regression to these data and data on 506 ADRs, producing 32 ADR-pathway associations supported by the scientific literature (*i.e.* PubMed). Second, Xie *et al.* [30] developed a methodology that identified 3D protein structures in the PDB that had similar ligand binding sites to those of the primary targets of Cholesteryl Ester Transferase Protein (CETP) inhibitors. Subsequently, they applied molecular docking to help rank order the atomic-level interactions of the drugs with the putative off-targets. This analysis led to 204 structures with binding sites similar to CETP. This set of off-targets was then integrated into a network that included multiple metabolic signal transduction and gene regulation pathway constituents, drugs, and clinical outcomes. From this network, they were able to elucidate several ADRs known to be associated with the CETP inhibitors: the negative effect of Torcetrapib on blood pressure observed in Phase III clinical trials and the increased death rates from infection and cancer.

These studies used the “first principles” approach to circumvent the bias issues in experimental data outlined above, but none of these previous efforts describe computational frameworks scalable to the data sizes required for a high-accuracy, high-throughput ADR screening panel for nascent compounds.

More recently, Reardon [31] reported on a computational effort that uses publicly available profiles of 600,000 chemical compounds and assesses their ability to bind to ∼7000 chemical pockets on 570 human proteins. The known expression profiles of the proteins and receptors on human organs is then used to predict where in the body a given drug will most likely have effects. While these efforts certainly operate at the necessary scale, they do not report a method to statistically associate the docking scores with ADR phenotypes, which is precisely the goal of our work here.

Our working hypothesis is that it is valuable to predict ADRs as early in the lead identification phase as possible. Structure-based, high throughput, virtual screening is already widely applied in the early stages of drug discovery because of its low cost and high efficiency in identifying putative drug-candidate/drug-target interactions. Molecular docking-based screening studies involve fitting a large library of N small molecules into the active sites of M target protein structures, to calculate estimates of binding affinities. M and N can be quite large. Currently, the PDB has M>90 K protein structures, increasing at a rate of over 7500 per year [26]. The combinatorics of the possible chemical structural space occupied by small molecules is immense, *i.e.* N ≈ 10^60^ possible drug compounds [32].

These numbers, combined with the complexities of conformational sampling to find the best fit of the small molecule (*i.e.* “pose”) in the target, and the computational cost of the scoring function itself, make high-throughput ADR screening ideal for high-performance computing.

Zhang *et al.* [33] implemented a mixed parallel scheme using Message Passing Interface (MPI) and multithreading in a parallel molecular docking program, called VinaLC, by modifying the existing AutoDock Vina molecular docking program. One million flexible docking calculations took about 1.4 hours to finish on ∼15 K CPUs. The docking accuracy of VinaLC has been validated against the DUD (Directory of Useful Decoys) database [34] by the re-docking of X-ray ligands and an enrichment study. The statistical results presented in their study [33] show VinaLC is one of the better performing docking codes on the DUD set of decoys/ligands, having a mean receiver operator characteristic area-under the curve (ROC AUC) of 0.64 (95^th^ CI: 0.60-0.68). VinaLC identified 64.4% of the top scoring poses with an RMSD under the 2.0 Å cutoff, while that for the best poses is 70.0%. For the best poses, all the targets have RMSD values within 10 Å and about half of the targets have RMSD values less than 1 Å. Overall, the VinaLC docking program performed well for re-docking the X-ray ligands back into the active site of the X-ray structures with the default setting for the grid sizes and exhaustiveness = 8. To improve the enrichment of the docking results, Zhang *et al.* [35] have also developed a massively parallel virtual screening pipeline using Molecular Mechanics/Generalized Born Surface Area (MM/GBSA) rescoring and have shown improvements in the docking benchmark AUC to 0.71, on average. Overall, the results demonstrate that MM/GBSA rescoring has higher AUC values and consistently better early recovery of actives than VinaLC docking alone.

A significant fraction of these molecules (*e.g.* drugs approved by regulatory agencies like the U.S. Food and Drug Administration) are annotated with known associated ADRs in public databases, such as SIDER. As in the prior work we cited, machine learning methods can identify statistical associations between these ADR outcomes and patterns in drug-protein binding as revealed by our VinaLC docking scores. The results can be used to build predictive models so the probabilities of certain ADRs can be predicted for a nascent or theoretical small molecule drug candidate that may not have undergone *in vitro* or clinical trial testing.

This study potentially provides a technological and methodological path forward to large-scale, high-throughput, *in silico*, comprehensive ADR screening. Our results indicate that molecular docking performed with sufficiently detailed docking models on high performance computers (HPC) may provide reliable, cost-effective, comprehensive high-throughput screening of a drug candidate for binding across many known on- and off-targets to predict clinically important ADRs.

## Materials and Methods

### Dataset creation

We extracted 4,020 Swiss-Prot protein knowledgebase UniProt ID numbers (http://www.uniprot.org/) for proteins that were identified as drug targets in DrugBank as of October 12, 2012 (http://www.drugbank.com/). Mappings to 587 experimental structures in the Protein Data Bank (http://www.rcsb.org/pdb/) (PDB) were obtained using the pdbtosp.txt file (Nov 2, 2013) from http://www.uniprot.org/docs/pdbtosp which links PDB ID numbers to UniProt IDs. A set of quality control rules were then applied (Figure S1 in File S1) which further reduced the list of proteins down to a final set of 409 experimental PDB structures. If multiple structures were given for the same protein, structures were selected by the following criteria in priority order: (1) human species; (2) X-ray crystal structure; (3) higher structural resolution (smaller Å). This set of PDBs included 33 structures belonging to 16 UniProt IDs that are a subset of a larger consensus *in vitro* toxicity panel. This panel consists of 44 targets that were presented as a minimum *in vitro* toxicology panel from a collaboration of four major pharmaceutical companies [6]. The structures of 906 FDA-approved small molecule compounds in SDF format were obtained from the “Orange Book” of approved products [36]. Drugs that have more than 20 rotatable bonds were not included because most of them are natural products. The 3D structures of target proteins and the small molecule compounds were then prepared for molecular docking calculations as described below.

A set of 85 side effects were selected from the SIDER database (http://sideeffects.embl.de/; extracted on November 26, 2012) because they were associated with high morbidity, high case fatality ratio, and/or the need for extended hospitalization. Individual side effects were grouped into higher-level health outcome groupings to reduce noise and provide signals at the organ or system level. Individual side effects were identified as lowest level terms in the medical dictionary for regulatory activities (MedDRA) [37]. Following the work of Huang and co-workers [25], the side effects of interest were grouped into ten MedDRA-defined system organ classes: (1) Neoplasms, benign, malignant, and unspecified (“neoplasms”), (2) Blood and lymphatic system disorders (“bloodAndLymph”), (3) Immune system disorders (“immuneSystem”), (4) Endocrine disorders (“endocrineDisorders”), (5) Psychiatric disorders (“psychDisorders”), (6) Cardiac disorders (“cardiacDisorders”), (7) Vascular disorders (“vascularDisorders”), (8) Gastrointestinal disorders (“gastroDisorders”), (9) Hepatobiliary disorders (“hepatoDisorders”), and (10) Renal and urinary disorders (“renalDisorders”). A subset of 560 of the 906 compounds in our docking score set were found to have associations to at least one of the 85 side effects we consider. The complete list of side effects by organ class is presented in Table S1 in File S1. We produce a 560×10 drug-ADR matrix where a ‘1’(‘0’) indicates the presence (absence) of one or more side effects in the group.

At the end of the dataset creation stage, we have a total of 906 compounds (560 with ADR associations), 409 proteins, and 10 outcome groups, comprising 85 severe side effects.

In order to compare the ADR prediction capability of “off-target” effects, obtained by the molecular docking calculations, with that of experimentally derived “on-target” drug-protein associations, a 560 drug×555 target protein association matrix was extracted from DrugBank. More precisely, in order for a specific protein to be in the list of 555 proteins, it must be identified as a ‘target’ in the DrugBank database of one or more of the 560 drugs in our dataset. The matrix is boolean-valued where a ‘1’(‘0’) indicates the presence (absence) of the association in DrugBank.

### Drug-protein target molecular docking calculations using VinaLC

The 409 target protein structures retrieved from the PDB were processed for molecular docking calculations. The raw PDB files were processed by our in-house Protein Function Prediction (PFP) pipeline [38]. The structures of the protein targets were cleaned and protonated. “Cleaning” was defined by the following: alternate location “a” records for atoms were kept, and any ligands (*i.e.* atoms designated as ‘HETATM’ after the TER record in the PDB file that are not part of common ions) were deleted. Molecular modeling software (Schrodinger Inc.) was used to protonate the protein structure. In those cases where a known catalytic site was identified, the centroid coordinates for the active sites/binding sites of the protein targets were determined by CatSId (Catalytic Site Identification) [39], otherwise, these sites were determined by Sitemap [40]. A similarity to a known catalytic site was identified in 83 cases. Cofactors, metals, and crystallographic waters were removed from the protein structure when performing the docking calculation [34], [41], [42], [43]. Missing residues in the active site were reconstructed. For structures with residues having multiple positions, the first one was used. These pre-treated protein target structures were further processed by the in-house program, preReceptor [35]. The program preReceptor provides interfaces to integrate several external programs for target protein preparation. The preReceptor program determines the dimensions of docking grids by utilizing the DMS [44] and SPHGEN programs [45]. The DMS program calculates the molecular surface of the target protein, and the SPHGEN program fills the active site of the target protein with non-overlapping spheres of uniform dimension. The dimensions of the docking grid for each protein were determined by first finding the distribution of spheres along the X, Y, and Z axes. The grid boundaries were set to the location where the density of spheres falls off drastically. In order to reduce the computer time, the docking grid determination was limited to portions of the target protein within 30 Å of the centroid of the active site (60 Å maximum diameter) because binding pockets typically are less than 40 Å in diameter. The dimensions of the docking grids and centroid of active site were stored for the docking calculation in the next step. The AMBER force field f99SB [46] was employed in the calculations. Non-standard amino acids distant from the binding site were converted to alanine. Otherwise, non-standard amino acids were stored in the library, if present in the active site. [47]. The energy minimization of the protein target was carried out using MM/GBSA [35] implemented in the Sander program of the AMBER package [46]. The structures were minimized with whole-protein heavy atom (*i.e.* all atoms that are not hydrogens) constraints so the geometry of the active site remains unchanged. The PDB files of energy-minimized protein structures were converted to PDBQT files, which are used in the docking procedure. During the conversion, the non-polar hydrogen atoms are removed from the protein target structures. Parameters for non-standard amino acids were calculated by the Antechamber program from the AMBERTOOLS suite. The set of 906 approved drugs were processed by the in-house program, preLigand [35]. Similar to the program preReceptor, the program preLigand provides interfaces to integrate several external programs for ligand preparation. All drug compounds were parameterized using the AMBER GAFF force field as determined by the Antechamber program in the Amber package [46]. Partial charges of ligands were calculated using the AM1-BCC method. The structures of ligands were energetically minimized by the MM/GBSA [35] method implemented in Sander. The atomic radii developed by Onufriev and coworkers [48] (AMBER input parameter igb = 5) were chosen for all GB calculations [49]. Those atoms with GB radii missing from the original program (*i.e.* fluorine, using a GB radius of 1.47 Å) were added into the Sander program. The PDB files of energy-minimized ligand structures were converted to PDBQT files, which were used in the docking procedure. As with the receptors, non-polar hydrogen atoms were removed from the ligand structures. All these steps mentioned above have been integrated into the preLigand program.

The VinaLC parallel docking program [33] was employed to dock the 906 drug compounds into the 409 protein targets. In our previous work [35], keeping 5–10 poses provided a good compromise between accuracy and computational expense. For each of the 906×409 = 370,554 individual drug-protein complex, docking calculations, up to 20 poses were kept. The docking calculations used the coordinates of centroids and dimensions of active sites determined from the previous steps. The PDBQT files for target proteins and compounds obtained from previous steps were used as input files. The docking grid granularity was set to 0.333 Å. The exhaustiveness was set to 12, so that 12 Monte Carlo searches for docking poses were performed for each complex. The whole calculation was finished within 1 hour on a high performance computer at LLNL using ∼15 K CPU cores. The top 20 docking poses were saved for each complex. The top docking score of each complex was extracted from the docking results. A table of docking scores for the 906 ligands×409 receptors, together with the compound’s PubMed ID/name and protein PDB ID, was saved in the CSV format for the statistical analysis described in the following section. Finally, we constructed a virtual version of the consensus toxicity-screening panel of 33 protein receptors. For this smaller 560×33 subset of scores, MM/GBSA [50]–[57] rescoring calculations were performed on the VinaLC docking poses. To achieve high throughput, molecular docking programs usually employ less computationally intensive methods such as molecular mechanics force-fields, empirical scoring functions, and/or knowledge-based potentials [57]. The scoring functions often simplify the calculation by neglecting important terms that are known to influence the binding affinity, such as solvation, entropy, receptor flexibility, etc [58], [59]. A popular practice – that we employ here – is to rescore top-ranking docking poses using the more accurate, albeit computationally costly, MM/GBSA method to overcome shortcomings in the docking scoring function [35]. The MM/GBSA method accounts for solvent and entropy effects more accurately. Solvation effects, mainly contributed by water molecules in biological systems, play a critical role in ligand binding by providing bulk solvent stabilization and solute-desolvation, increasing the entropic contribution with the release of water molecules in the active site upon binding, and serving as molecular bridges between the ligand and receptor [58].

The protein structures and docking co-complex structures are downloadable at http://bbs.llnl.gov/data.html. The VinaLC software package can be downloaded from http://bbs.llnl.gov/tools.html.

### Statistical analysis

The molecular docking calculations produced a 906×409 drug-protein docking score matrix. A 560×409 subset was extracted, where each of the 560 compounds has at least one side effect, as reported in SIDER, for the 10 ADR groups we are considering. Statistical analyses were performed on these data to train predictive models of serious ADRs and characterize putative ADR-protein associations. These analyses are outlined here.

For the analyses, four separate data matrices are considered: (A) a 560×409 VinaLC drug-protein docking scores matrix (“VinaLC off-targets”) and (B) a 560×555 DrugBank drug-target protein association matrix (“DrugBank on-targets”). Matrix (A) is used to train logistic regression models that allow off-target ADR-protein correlations to be explored. Matrix (B) is used to train models on “on-target” drug-protein associations. The comparison of results between matrices (A) and (B) enables comparisons to be made between the relative predictive capabilities of intended target proteins and off-targets across the different ADR groups. The 16 toxicity panel target proteins in isolation are considered; thus, we also have (C) a 560×16 docking score matrix which is a subset of (A) and finally (D) a 560×16 boolean matrix which is analogous to (B), representing any drug-target associations reported in DrugBank between the 560 compounds and the 16 proteins of the toxicity panel. It is noted that the separate matrices (C) and (D) are constructed for the same on-target/off-target comparison purpose as matrices (A) and (B). Regarding the construction of the (C) matrix, there were 33 structures for the 16 proteins. Thus, multiple PDB structures that mapped to the same UniProt ID were averaged over, so (C) and (D) matrices are conformable. We note here that this was only done for the virtual toxicity panel. For the main VinaLC docking score matrix (A), the scores for individual PDB structures were mapped one-to-one to the relevant UniProt ID for that protein. The elements in matrices (B) and (D) also correspond to single UniProt IDs.

Next, we define thresholds so the docking scores in matrices (A) and (C) can be used as a heuristic for drug-protein binding. Global and protein-specific thresholds are defined. The raw docking score itself is used as a continuous feature, and (given that more negative scores correspond to stronger binding) additional thresholds are defined such that a docking score below the threshold indicates binding or, if above it, not binding. The docking score does not correspond to an actual energy, and it is difficult to set a single value for a threshold. Several thresholds are tried, letting the quality of the models (as quantified by the AUC) determine the best threshold for each ADR. For the VinaLC scores, ten feature sets are used, based on different choices of threshold: (1) raw docking scores, and then a series of global binding cutoffs: (2) -4.0, (3) -6.0, (4) -8.0, (5) -10.0, and (6) -12.0. Four additional thresholds based on protein-specific score percentiles were also defined: (7) 5th percentile, (8) 10th percentile, where the percentiles refer to the docking scores across all 560 compounds for a given protein. The last two thresholds were calculated by transforming the 560 docking scores for each protein into z-scores (*i.e.* transformed to have zero mean and unit standard deviation). Thresholds of (9) 1 standard deviation (SD) below the mean score (as used in the docking studies of Wallach and co-workers [29]) and (10) 2 SDs below the mean are also used. For the 560×16 virtual toxicology panel, which used GBSA scores, the global thresholds were -15, -20, -25, -30, and these can be interpreted as binding free energies. Raw scores, protein-specific percentiles, and z-score thresholds are used as features, analogous to the thresholds defined for the VinaLC score matrix (A).

Logistic regression models were trained and selected through 10-fold cross-validation (CV) applied to the ten feature sets each for the data matrices (A) and (C) and then for the Boolean matrices (B) and (D). The training samples were labeled by the 560×10 response matrix, consisting of the Boolean associations between the 560 compounds and the ten ADR groups, leading to 22 separate CV runs in all.

The lasso penalty or L1 model regularization [60] is an effective method for continuous variable selection in the regime where the number of potential features is comparable to (or may actually exceed) the number of training samples (*i.e.* 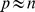 where *p* is the number of potential predictor variables, and *n* is the number of training samples). The L1 penalty term is proportional to the sum of regression coefficients 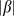 that fall off faster than the 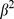 terms used in L2 regularization for small values of beta, so the lasso penalty is efficient at shrinking the betas to exactly zero, enabling sparse solutions and thus greater interpretability. The sparseness makes this method especially effective in the biological domain, where frequently a much smaller subset of the features can explain the phenotype or outcome. L1 logistic regression has been successfully applied to single nucleotide polymorphism (SNP) analysis [61], as well as previous ADR prediction studies [29].

The ADR prediction problem considered here can be formalized as a case-control problem where a dichotomous variable 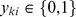 is defined for the i-th sample and k-th ADR health outcome group with ‘1’ coding cases and ‘0’ indicating controls. Given a feature vector for the i-th sample, 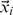, the probability for the k-th outcome is given by
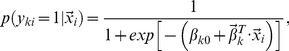
(1)where 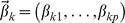 is the parameter vector (and 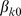 is the intercept term) for the k-th outcome, and is typically estimated by maximizing the log-likelihood function
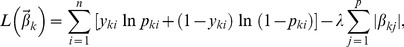
(2)where the second term in Eq. (2) is the lasso penalty.

The L1-regularized logistic regression was used as implemented in the glmnet package of Friedman and co-workers [62] in the ‘R’ statistical programming environment. For each of the 10 ADR outcome groups in turn, one-vs-all logistic regression was used with 10-fold cross validation. During 10-fold cross validation, the following was done simultaneously: the objective function (AUC) was maximized, the model parameters in Eqs. (1) and (2) were estimated, and the optimal L1 penalty parameter in Eq. (2) was chosen as the one corresponding to the maximum median AUC. Each 10-fold CV was repeated ten times to average over sampling variability.

After 10-fold CV, for each of the four data matrices (A)–(D), features with non-zero beta coefficients in the best median AUC model were extracted. The statistical significance of putative associations between the ADR groups and docking score matrix protein features were calculated. Statistical significance of the association for a putative ADR-protein pair was determined by the following procedure: univariate p-values for each ADR-protein pair were calculated using Fisher’s exact test if the protein feature was dichotomous (*i.e.* associated with a binding threshold, or DrugBank association). If the feature was continuous (*i.e.* the raw docking scores), the Wilcoxon rank sum test was used. In addition to p-values, we analyzed the false discovery rate (FDR) due to multiple hypothesis testing. For the models associated with the larger Vina off-targets matrix (A), we calculated q-values, using the ‘qvalue’ R-package of Storey [63], which gives us a way to manage the high false discovery rate that can be associated with large feature sets. For the smaller virtual toxicity MM/GBSA matrix (C), the FDR was managed by applying a simple Bonferroni correction [64] to the p-value.

**Figure 1:**
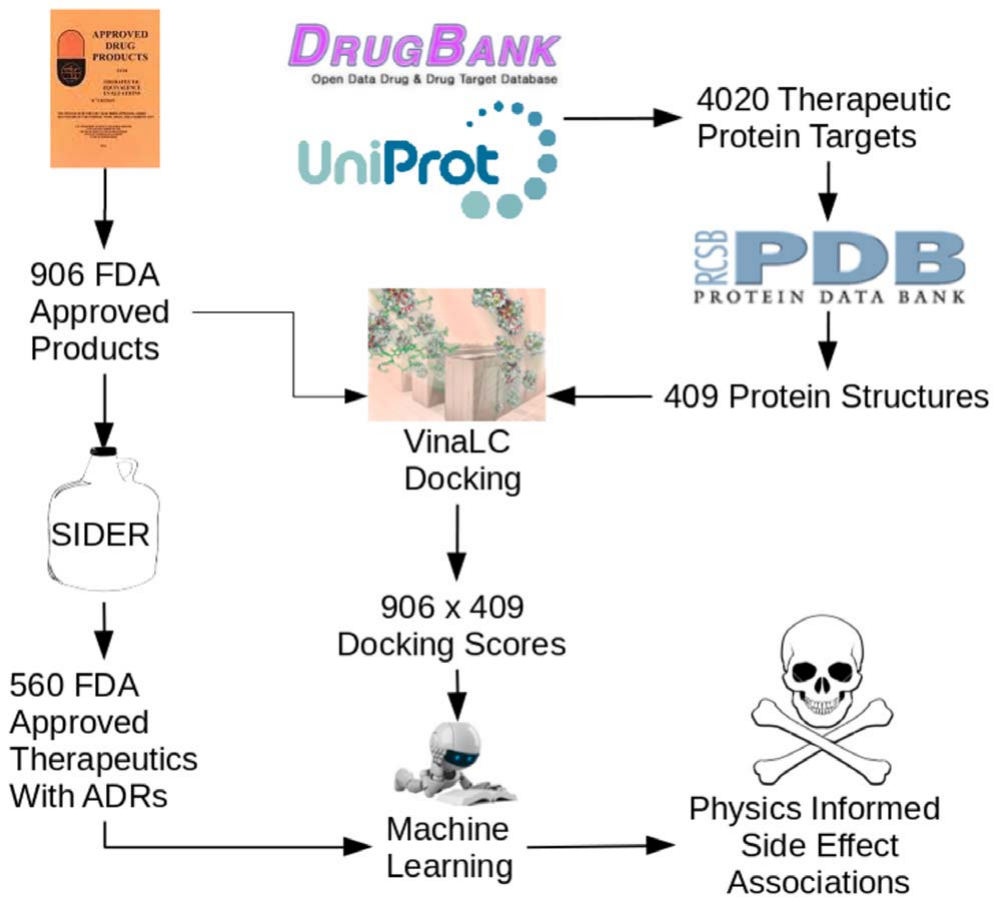
Data integration/analysis workflow scheme. The UniProt IDs of 4,020 proteins identified in DrugBank as drug targets were extracted. We obtained 409 experimental protein structures from the Protein Data Bank (PDB) to be used as a virtual panel and docked to 906 FDA-approved small molecule compounds using the VinaLC docking code, run on a high-performance computing machine at LLNL. 560 compounds had side effect information in the SIDER database and were used in subsequent statistical analysis to build logistic regression models for ADR prediction.

The workflow just described, comprising data integration between DrugBank, UniProt, PDB, and SIDER, as well as our docking score calculations and subsequent statistical analyses, is shown schematically in Figure 1. R scripts created and used for these analyses can be downloaded from http://bbs.llnl.gov/data.html.

### PubMed text mining to find supportive evidence of ADR-protein associations

PubMed database (http://www.ncbi.nlm.nih.gov/pubmed) queries were used to search for evidence in the literature to support putative ADR-protein relationships identified by the statistical analyses of the VinaLC drug-protein docking matrix. The protocol for searching the PubMed database was as follows: 1) Queries for co-occurrences of the UniProt name of the protein and the MedDRA lowest-level term (LLT) of each individual side effect constituent of the ADR group were performed, 2) If the number of hits returned was substantive (∼10), or the quality of the hits was high, then the association was triaged for manual review of the PubMed results set. An example of a high-quality hit is the side effect and the protein terms co-occurring in the title or abstract of an article. ADR-protein associations that passed the manual review process were deemed significant and included in Tables 1 and 2.

**Table 1:** Top-ranked ADR-protein associations derived from models built using the 560×409 docking score matrix.

UniProt Name	UniProt ID	PDB ID #	p-value	q-value	beta	UniProt protein-MedDRA side effect PubMed hits
Interstitial collagenase	P03956	1hfc	0.004	0.531	2.348	breast neoplasm(158), adenocarcinoma(161), glioma(34), basal cell carcinoma(22)
Tyrosine-protein kinase SYK	P43405	1xbb	0.012	0.531	1.213	breast neoplasm(46), adenocarcinoma(11)
Peroxisome proliferator-activated receptor alpha	Q07869	2znn	0.016	0.531	0.602	breast neoplasm(95), adenocarcinoma(146), glioma(25), basal cell carcinoma(14)
Complement C3	P01024	2wy8	0.034	0.531	0.698	breast neoplasm(65), adenocarcinoma(136), glioma(21), lung neoplasms malignant(12), basal cell carcinoma(7)
Cytotoxic T-lymphocyte protein 4	P16410	3osk	0.003	0.555	0.211	sarcoidosis(11), vasculitis(24)
Profilin-1	P07737	1fil	0.000	0.005	0.338	endocrine disorder(10)
Coagulation factor IX	P00740	1edm	0.000	0.005	0.019	endocrine disorder(108), diabetes mellitus(48), thyroid disorder(22), hyperthyroidism(11), hypothyroidism(10)
Interleukin-5	P05113	1hul	0.000	0.005	0.092	endocrine disorder(35), diabetes mellitus(19), thyroid disorder(10)
Caspase-3	P42574	2dko	0.002	0.188	−1.876	bipolar disorder(14), schizophrenia(31)
Integrin beta-2	P05107	2p26	0.020	1.000	−0.886	cardiac arrest(11), cardiomyopathy(44), myocardial infarction(46)
Interstitial collagenase	P03956	1hfc	0.000	0.060	0.429	aneurysm(39), aortic aneurysm(31), arteriosclerosis(123)
Gelsolin	P06396	2fh1	0.000	0.009	−0.073	nephropathy(38), renal failure(12)

The docked protein responsible for the association with the ADR is identified in the first, second, and third columns, using the UniProt name and ID and the corresponding PDB ID, respectively. Columns 4,5, and 6 give data on the statistical significance of the association with the p-value of the association, the associated false discovery rate (q-value), and the corresponding beta coefficient in the median AUC logistic regression model. Column 7 is the PubMed results that confirm the drug-protein or drug-side effect. The number of hits is shown in parentheses. Bold UniProt IDs are off-target proteins (*i.e.* not intended targets of the 732 drugs we consider).

**Table 2:** ADR-protein association derived from models built using the 560×16 GBSA-corrected virtual screening panel.

UniProt Name	UniProt ID	Corrected p-value	ADR Group	UniProt protein - MedDRA side effect PubMed hits
Amine oxidase [flavin-containing] A	P21397	0.005	bloodAndLymph	agranulocytosis(5)
Histamine H1 receptor	P35367	0.007	bloodAndLymph	agranulocytosis(10)
Beta-2 adrenergic receptor	P07550	0.007	endocrineDisorders	endocrine disorder(164), diabetes mellitus(98), thyroid disorder(31), hyperthyroidism(19), hypothyroidism(16)
5-hydroxytryptamine receptor 1B	P28222	0.007	endocrineDisorders	endocrine disorder(15), diabetes mellitus(11)
Androgen receptor	P10275	0.018	psychDisorders	schizophrenia(18)
Prostaglandin G/H synthase 2	P35354	0.024	cardiacDisorders	cardiac arrest(11), cardiomegaly(22), cardiomyopathy(91), myocardial infarction(217), myocarditis(11)

## Results

**Figure 2:**
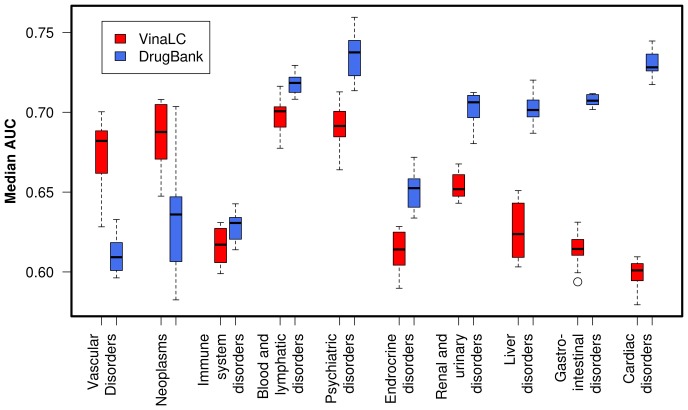
ADR prediction models using ‘Vina Off Targets’ and ‘DrugBank On-Targets’. Boxplots of median AUC results for one vs. all L1-regularized logistic regression models trained using 10-fold cross-validation repeated ten times are shown. The individual models were trained on ten different adverse drug reaction (ADR) groups: Vascular disorders ("Vascular disorders"), Neoplasms, benign, malignant, and unspecified ("Neoplasms"), Immune system disorders ("Immune system disorders"), Blood and lymphatic systems disorders ("Blood and lymphatic disorders"), Psychiatric disorders ("Psychiatric disorders"), Endocrine disorders ("Endocrine disorders"), Renal disorders ("Renal & urinary disorders"), Hepatobiliary disorders ("Liver disorders"), Gastrointestinal disorders ("Gastrointestinal disorders"), and Cardiac disorders ("Cardiac disorders"). Red boxes indicate models trained on 560×409 VinaLC docking scores used as drug-protein binding features. Blue boxes indicate models trained on a 560×555 matrix containing DrugBank drug-target protein associations. VinaLC off-target models had higher AUCs than DrugBank on-target models for the “Vascular disorders” and “Neoplasms” ADR groups.

The 560×10 drug vs ADR group matrix (C) and the 560×409 drug vs protein docking score matrix (A) were used to train logistic regression models using L1-regularization, which allows the model to focus on high-information predictors and helps reduce over fitting. Figure 2 presents the performance profile of our ADR prediction models. For each ADR group, a “best model” was chosen based on the median AUC score of a model obtained during a single ten-fold cross-validation run. The quality of these models was compared to models trained on the 560 drug×555 DrugBank on-target protein matrix (B), using the identical statistical model training procedure that was applied to the 560×409 VinaLC off-target docking score matrix (A). Figure 2 also compares the performance profile of the docking score models with that of the models trained on the DrugBank data. Across all ADR groups, the range of the best model AUCs for the VinaLC off-target models was 0.60–0.69. The corresponding AUC range for the DrugBank on-target models was AUC = 0.61–0.74. Focusing on single ADRs, the inter-quartile range of the VinaLC off-target AUCs is above those of the DrugBank on-target models for both ‘neoplasms’ and ‘vascularDisorders’ ADR groups. The AUC distributions are not significantly different between the two datasets for ‘immuneSystem’ and ‘bloodAndLymph’. The DrugBank model AUCs were larger for these ADR groups: ‘psychDisorders’, ‘endocrineDisorders’, ‘renalDisorders’, ‘hepatoDisorders’, ‘gastroDisorders’, and ‘cardiacDisorders’. The difference in AUCs implies the importance of the on-target binding contributions for the latter subset of ADRs.

The ability of docking score data to identify potential associations between off-target drug-protein binding and individual side effects in the ADR groups was investigated. Additional statistical analysis was performed on the VinaLC drug-protein docking score matrix and the logistic regression models to derive associations between ADR groups and proteins. Only 21% (87 out of 409) of the drug-protein binding features involve known protein targets of the drug subset, providing a significant probe of off-target effects. In Table 1, side-effect protein pair-wise associations are shown rank-ordered in ascending order, according to that feature’s p-value. For each entry we list the UniProt name and ID of the drug-binding protein, the PDB ID for the protein target used in docking, the p-value, the corresponding q-value to indicate the FDR for that feature, and the beta coefficient in the “best” model. Furthermore, the variable selection capacity of L1-regularization was employed, so that a protein feature must have a non-zero beta coefficient in order to have been included in Table 1. Finally, for inclusion in Table 1, the ADR-protein association needed to pass the manual review of PubMed evidence. In the last column of Table 1, the level of evidence from PubMed that supports the ADR-protein correlations is shown. For a specific putative ADR-protein entry in Table 1, counts in parentheses show the number of papers found in PubMed that contain the co-occurrence of (1) the MedDRA lowest level term for a component individual side effect from the ADR group and (2) the UniProt name for the protein.

**Figure 3:**
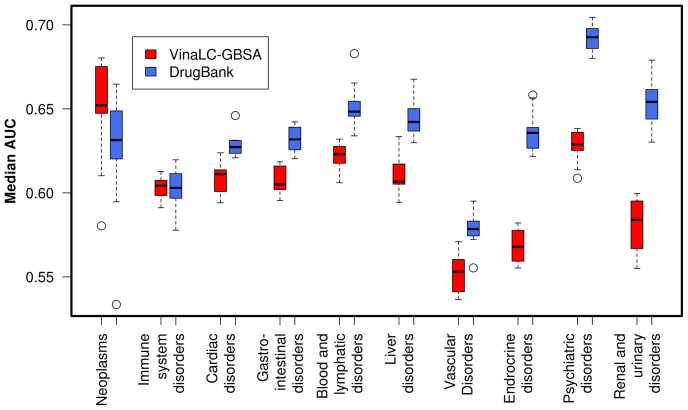
ADR prediction using a 16-protein virtual toxicity screening panel suggested by Bowes *et al.* [6]. Red boxes indicate models trained on GBSA-corrected VinaLC docking scores while the blue boxes indicate models trained on DrugBank drug-target protein associations. The boxplots comprise the distribution of median AUC scores after one vs. all L1-regularized logistic regression model training using 10-fold cross-validation repeated ten times. The individual models were trained on ten different adverse drug reaction (ADR) groups: Neoplasms, benign, malignant, and unspecified ("Neoplasms"), Immune system disorders ("Immune system disorders"), Cardiac disorders ("Cardiac disorders"), Gastrointestinal disorders ("Gastrointestinal disorders"), Blood and lymphatic systems disorders ("Blood and lymphatic disorders"), Hepatobiliary disorders ("Liver disorders"), Vascular disorders ("Vascular disorders"), Endocrine disorders ("Endocrine disorders"), Psychiatric disorders ("Psychiatric disorders"), and Renal disorders ("Renal & urinary disorders").

The associations between the ten ADR groups and a subset of the full VinaLC off-target docking score matrix (C) were investigated. Models trained on a 560×16 subset of the full VinaLC docking score matrix (C) were compared to the models trained on a 560×16 DrugBank on-target subset matrix (D). The docking calculations were refined using the more computationally expensive and more chemically accurate MM/GBSA-correction of the Vina score. The same logistic model training procedure used on the larger predictor sets to train logistic regression models was applied to these smaller matrices. The boxplots of the “best model” AUCs for the screening panel models are shown in Figure 3. Overall, the range of AUCs for the MM/GBSA “off-target” version of the consensus panel (AUC = 0.55–0.65) and the DrugBank “on-target” version (AUC = 0.58–0.69) of the panel indicate that the quality of the models are only marginally poorer than those derived from the larger predictor set, but use a factor of ∼26 fewer protein features, indicating they may have some value in the drug development pipeline.

Across the ADR groups, the MM/GBSA and DrugBank virtual panel model AUCs are similar for ‘immuneSystem’, ‘cardiacDisorders’, ‘gastroDisorders’, ‘bloodAndLymph’, and ‘hepatoDisorders’. The MM/GBSA-derived models for the ‘endocrineDisorders’, ‘psychDisorders’, and ‘renalDisorders’ ADR groups are all significantly worse than the corresponding DrugBank models.

Given the role of these 16 proteins in *in vitro* toxicity panels, it is of interest to see what specific associations they may have with specific side effects. Potential ADR-protein associations are shown in Table 2, listed by UniProt name and ID. All potential ADR-protein associations had to have a Bonferroni-corrected p-value < 0.05 and a non-zero beta coefficient in the “best” logistic regression model. Additionally, the associations had to pass the same manual review process used for the associations listed in Table 1.

## Discussion

The major contribution of this work is a demonstration of the feasibility to holistically treat the ADR prediction problem for nascent drug compounds. Our methods treat the problem from atomistic levels (*i.e.* drug-protein binding) all the way up to prediction of clinical ADR phenotypes. We show, for our particular set of 560 drugs, that using molecular docking scores yields ADR prediction models comparable in quality (as evaluated by AUCs) to models developed using publicly available, experimentally-derived drug-protein associations. However, the AUCs, for both docking scores and experimental data, are not of sufficient quality for clinical prediction, and it is interesting to note the quality is poorer for highly multi-factorial disorders (*e.g.* cardiac disorders). As an example, for the virtual toxicity panel model quality results shown in Figure 3, we can see that for psychological disorders, the on-target relationships in the virtual panel yield a model with AUCs close to 0.7, while the MM/GBSA-rescored docking scores, emphasizing off-target effects, yield an AUC slightly better than random (*i.e.* AUC = 0.5).

We first discuss some issues related to the molecular docking score calculations. The accuracy of docking calculations [65] and in particular, how to best account for explicit water placement [58], [59], [66], [67] and metal center interactions [68]–[70], remains an open question outside the scope of this work. Our calculations examine the binding of drug ligands to off-target proteins, where typically little or no data exists to inform initial placement of water molecules, metal ions, co-factors, or other hetero atoms. Protein flexibility is yet another issue we neglect. Without a generally accepted protocol to deal with all of these issues in an automated fashion, we took the simplest approach and removed all water molecules and co-crystallized ligands from the protein crystallographic structures in preparation for docking. The docking calculations could be more accurate in the final poses and estimated energies if explicit waters, metal ion chelation interactions, and flexibility were explicitly addressed. However, despite the shortcomings of the docking calculations, statistically distinguishable correlations between the docking results and ADRs are still observed. We would expect that with improved binding estimates, the statistical correlations should improve and that the results from this work will motivate and justify similar efforts using more sophisticated techniques for computing binding affinities.

As stated in the Methods section, several different binding thresholds for the docking scores were tried. Both the VinaLC and MMGBSA logistic regression model AUCs did not monotonically vary with choice of thresholds, and there were no clear trends with threshold choice. However, we did observe that in the ADR groups with lower overall AUC values, the maximum AUC among the different threshold values was, in some cases, only greater than the second largest AUC by a few ∼0.01. For ADR groups with the best AUCs, the maximum AUC was often more clearly differentiated from the AUCs of the other competing threshold values.

Models trained to predict side effects in the ‘neoplasms’ and ‘vascularDisorders’ ADR groups on the full 560×409 VinaLC docking score matrix (A) perform better than their DrugBank-derived counterparts (B).

We identify several potential off-target ADR-protein associations that would be impossible to find using only binding data between a drug and its intended protein targets (see Table 1). Some of the more compelling associations found are described below, along with supporting evidence from the literature. The literature cited here may describe examples where biological mechanisms are perturbed by drug binding to protein constituents of pathways associated with the ADRs.

### Interstitial collagenase (MMP1) with both neoplasms and vascular disorders

Increased MMP-1 gene expression appears to be a biomarker for cancer metastasis. Specifically, we find evidence for separate constituents of the ‘neoplasms’ group: breast neoplasms [71], adenocarcinoma [72], and glioma [73]. Interstitial collagenase also seems to contribute to aneurysms. Specifically, cell distribution differences of MMP-9 and the tissue inhibitor of MMP-1 in patients with Kawasaki disease [74]. This work implicates interaction of MMP-9 and MMP-1 with aneurysm formation in Kawasaki disease.

### Tyrosine kinase Syk with breast neoplasms and adenocarcinomas

A possible mechanism of interaction may be a role in suppression of breast cancer metastasis to lymph nodes [75], as well as regulating cell-cell adhesion and motility [76]. Some data suggest that Syk expression in the spleen may inversely correlate with the proliferation and invasive capacity of breast cancer [77]. Syk acts as a pancreatic tumor suppressor in pancreatic adenocarcinoma tumors, regulating cellular growth and invasion [78].

### Complement C3 with breast neoplasms

An analysis [79] of expression patterns for acute phase proteins in breast, colorectal, and lung cancer indicate that the most accurate candidate biomarker for breast cancer in their panel was Complement 3 (C3) as used in a univariate logisitic regression model (AUC = 0.89 and 73% correct classification performance in leave one out cross-validation).

### Cytotoxic T-lymphocyte protein 4 (CTLA-4) with sarcoidosis

A case study [80] shows exacerbation of sarcoidosis in a melanoma patient treated with anti-CTLA-4 monoclonal antibody inhibitor ipilimumab. Another study [81] reports correlations of specific CTLA-4 gene polymorphisms in sarcoidosis patients with different disease phenotypes.

### Profilin-1 with endocrine-related disorders

Profilin-1 expression is markedly elevated in the atherosclerotic plaques of diabetics, showing a potential role in mediating diabetic-related vascular endothelial cell dysfunction [82].

### Coagulation factor IX with thyroid disorders

A meta-analysis [83] looked at 29 trials and 11 studies and concludes that subclinical hyperthyrodism induces a pro-thrombotic state. More precisely, thyrotoxicosis shifts balance to a pro-coagulant/hypofibrinolytic state.

### Caspase-3 with bipolar disorder and schizophrenia

Some papers hypothesize that enhanced cellular apoptosis is a disease mechanism in neurodegenerative diseases. A postmortem study on bipolar disorder patients shows significant increases in pro-apoptotic factors (inc. Bax, BAD, caspase-9 and caspase-3) [84]. A population of anti-psychotic medicine-naive first-episode schizophrenia patients show higher caspase-3 activity and lower BCL2 expression [85].

### Integrin beta-2 and myocardial infarction

Studies have shown integrin and monocyte migration are associated with ischemic myocardium. A study [86] that performed flow cytometry-based whole-blood assays in 87 patients with unstable angina finds that beta-2 integrin mediated T-cell recruitment in coronary plaques identifies high-risk patients with severe coronary artery disease but no myocardial infarction and is predictive of future cardiovascular events, even in the absence of myocardium damage markers like troponin or high-sensitivity C-reactive protein.

We also find ADR-protein associations for the 16-protein consensus panel. The protein targets are included in panels used by major pharmaceutical companies for *in vitro* screening of ADRs for drugs in the development pipeline. Our results provide a rationale, founded on independent calculations, for their inclusion in the panel based on side effect phenotypes for which they probe. Potential ADR-protein associations, supported by some level of evidence in PubMed, are listed in Table 2. Among them, we found a correlation between agranulocytosis and the histamine H1 receptor (an example is the drug clozapine an H4-receptor agonist with some H1 activity) [87]. Also, a number of cardiac-related side effects were associated with Prostaglandin G/H synthase 2 (Cyclooxygenase 2), in particular ‘myocardial infarction’ which yielded 217 PubMed hits.

Using molecular docking scores for drug-protein matrices has advantages over other approaches to predict association of off-target effects. Molecular docking is an approach based on a physics-derived force field, such that only the structure of the drug and the protein are necessary. Not surprisingly, the docking approach does not have as strong a dependence on the availability of drug-protein correlations in manually curated biological or chemical databases (which are biased toward intended, on-target effects), though these data can be integrated into our type of analyses as well. Experimental drug-protein association matrices are extremely sparse, *i.e.* there are large areas of the drug × protein matrix that are unexplored by *in vitro* assays or clinical trials. In contrast, the docking calculations enable an exhaustive probing of binding associations through the entire drug × protein matrix, allowing the exploration of unintended (*i.e.* off-target) interactions that may not have been previously experimentally investigated during drug development. Thus, docking scores provide a direct way to probe off-target effects.

Here we compare our work to previous efforts that have applied molecular docking to study ADR-protein correlations. A recent large-scale drug-protein docking exercise was described by Reardon [31], but this effort had a different goal than our study. While the work outlined in [31] appears to focus on a highly automated method where structures are prepared and docked in a bulk fashion, we have chosen to initially focus on a smaller group of drug-protein interactions, hand-curating the initial docking structures, so the quality of the drug-protein binding is sufficiently high that we can link to ADR outcomes downstream of docking. In [31], it appears that no attempt, beyond identifying the tissue tropism of the receptors used in docking, is made to correlate the results of docking to ADR phenotypes. The work of Wallach *et al.* [29] bears some similarities to our work, and here we list some of the major differences between the two efforts. Specifically, we: 1) use q-values to correct for multiple hypothesis testing, which has been previously shown to indicate “interesting” protein-side effect correlations [21], 2) focus on proteins rather than pathways and on only on a small set of serious ADRs, 3) consider multiple binding thresholds for binding, in addition to one standard deviation above the mean of the z-scored docking scores used by Wallach *et al.*, 4) compare model performance across ADRs (thru AUC), where the work in [29] is focused on ADR-pathway associations, and 5) are interested in ADR prediction using the docking scores. Although, Wallach *et al.* also use L1-regularization to mitigate over-fitting, our lambda parameter is chosen through 10-fold cross-validation, while their lambda parameter seems to have been arbitrarily chosen to be one-half the value needed to suppress all beta coefficients to zero. They do not appear to discuss the associations they produce in quantitative terms (e.g. AUCs of the models, p-values of the associations). Also, their study treats each side effect individually, which may lead to bad class imbalances with more rare ADRs, a common problem in QSAR studies. We mitigate this issue by classifying ADR phenotypes into groups.

Another study similar to ours is the work of Xie and co-workers [30]. As in our study, they utilized an algorithmic approach to find previously unidentified binding sites on putative target proteins. They then applied a serial version of AutoDock to characterize interactions between drugs and off-target proteins and evaluate the viability of the proteins as off-targets. These data were then combined with pre-existing data in the biological/medical literature to place their findings into a larger context. That is where the similarities end. The main focus of [30] was to deduce ADR mechanisms for a single class of drugs (*i.e.* CETP inhibitors). Candidates for off-targets were limited to those proteins that had binding sites with a high-degree of similarity to the CETP binding site. The focus for our study was not a careful, detailed study of a particular system, but rather the development of a general tool that can look across multiple drug classes and a heterogeneous mixture of off-target proteins. Our methods provide a way to obtain correlations between the molecular details of docking and several clinical phenotype groups of ADRs going across several organ systems. The work of Xie *et al.* also provides linkages to ADR phenotypes, but spends much more effort at understanding the mechanistic details of “meso-scale” biological pathways, and how they bridge the binding event to downstream signaling and gene regulation events that may be the actual causative factors that give rise to CETP inhibitor-relevant ADRs. This detailed work must be done to achieve true understanding of how a drug causes an ADR. Their approach may offer a possible template for how to integrate docking studies with systems biology approaches, but it is difficult to see how one could scale-up their approach to a more general-purpose ADR-protein correlation tool such as ours. Combining multiple off-target effects at the pathway level would be a worthwhile improvement to our methodology.

The limitations of our method can be categorized into two areas: 1) molecular docking and 2) ADR phenotypes. For molecular docking to be a feasible method for predicting “off-target” associations, the execution of the docking needs to be fast and reliable. Our implementation of the well-vetted Vina docking program, VinaLC, has been optimized for HPC and has been benchmarked with known limitations (*e.g.* metalloproteins) [33]. We noted the inherent biases in the QSAR-like studies given their reliance on experimental data derived from approved drugs. While the molecular docking study advocated here does not suffer the same bias towards approved drugs, the docking methods are heavily biased toward proteins that have available 3D structures, which restricts these molecular docking to ∼50% of the human proteome, as estimated by Xie *et al.* [30]. In addition, of the 3D structures that are available, we are still limited by the number of target proteins associated with relevant side effects. Unfortunately, the missing cohort of proteins will be highly enriched with some of the more important classes for ADRs, namely membrane-bound receptor proteins. With the growing number of protein crystal structures and the higher quality homology models, the availability of quality 3D protein structures is growing each year. In principle the docking score technology and statistical analyses methodology can scale to large numbers, but the actual scaling behavior has yet to be characterized. As new proteins and new drugs are added to our calculations, we would expect quadratic scaling in the drug × protein matrix. The machine learning algorithms used to learn statistical correlations from this data should scale as a higher-degree polynomial of the number of training samples, *i.e.* docking profile of a drug. The benefit of using an HPC platform is that the effects of non-linear scaling can be addressed by the allocation of additional compute nodes and processors. Investigation of the actual scaling behavior with increasing data set size and increasing number of CPUs remains to be done as future work.

For ADR phenotypes, we are currently limited by the availability of clinical data on ADR phenotypes linked to drugs. In addition, publicly available ADR outcomes data will always be biased toward approved drugs. Our results point to the importance of the target proteins, which might not be known for nascent compounds. The known or intended targets appear to be important for ADRs associated with major organ systems (*e.g.* renal, hepatic, and cardiac). Results of the toxicity panel analysis indicate that even at the MM/GBSA level, we need to improve the drug-target interaction estimates, as shown by the poor performing median AUCs for the ADR groups endocrine, psychiatric, and renal. Also, the minimal "comprehensive" set of proteins needed to obtain high-quality ADR prediction models is unknown. As more proteins and pathways are associated with ADR phenotypes, the minimal comprehensive set will be soon be obtained.

Limitations associated with the way putative ADR-protein associations are corroborated with literature studies may also exist. Biological terms are used ambiguously in the literature. Our intent was to find a well-defined (*i.e.* UniProt names for proteins and MedDRA lowest-level terms for side effects), standardized way to see a preponderance of papers (*e.g.* more than 10) in the literature, where a sample could be obtained and examined manually for the quality of the correlation. In no way are we reporting exhaustive numbers of papers that contain a particular putative ADR-protein correlation in PubMed. Any other approach (*e.g.* stemming the terms) would also have some ambiguity associated with it.

## Conclusions

We have shown in this study that molecular docking may enable reliable, cost-effective, comprehensive, high-throughput screening of a drug candidate for binding across many known targets to provide predictions of clinically important ADRs. We introduce a first principles approach to *in silico* ADR prediction for drug compounds that leverages physics-based models and HPC by docking 560 small molecule drugs to 409 structures of identified DrugBank protein targets. Only 21% (87 out of 409) of the drug-protein binding features involve known targets of the drug subset, providing a significant probe of off-target effects. The median AUCs obtained during 10-fold cross-validation were comparable between the VinaLC off-target models (AUC = 0.60–0.69) and the DrugBank on-target models (AUC = 0.61–0.74) across the ten ADR groups. Most importantly, the VinaLC off-target model out performed the DrugBank on-target model for predicting two ADR groups, neoplasms and vascularDisorders. We further investigated the associations between the ten ADR groups and a consensus subset of 16 proteins used in early-stage *in vitro* toxicity screening panels. The analysis identified several putative ADR-protein associations. Successful PubMed queries found published results in support of these putative ADR-protein associations. For example, several associations between neoplasm-related ADRs and known tumor suppressor (Syk) and tumor invasiveness marker (MMP-1 and C3) proteins are found. Many of these associations involve off-target proteins and would not have been found using only the available drug-target data. Thus, increasing the reliability of the drug-protein binding calculations and increasing the protein target set to include more proteins outside the known protein targets in DrugBank should identify additional off-target proteins that are associated with possible ADRs. This predictive computational platform would be advantageous during drug development to predict ADRs of drug candidates such that candidates could be dropped or redesigned at an earlier stage.

## Supporting Information

File S1 Figure S1, Protein structure quality control workflow used in preparation for docking calculations. Table S1, ADR groupings and their MedDRA LLT side effect components.
(DOCX)
