# Supplementary material for: Adverse Drug Reaction Prediction Using Scores Produced by Large-Scale Drug-Protein Target Docking on High-Performance Computing Machines
Source: PLoS One. 2014 Sep 5;9(9):e106298. doi: 10.1371/journal.pone.0106298 (PMC4156361; doi:10.1371/journal.pone.0106298)
Supplement: File S1 — Figure S1, Protein structure quality control workflow used in preparation for docking calculations. Table S1, ADR groupings and their MedDRA LLT side effect components. (DOCX) [file pone.0106298.s001.docx]

(S1) SUPPLEMENTARY INFORMATION FOR:

**“Adverse Drug Reaction Prediction Using Scores Produced by Large-Scale Drug-Protein Target Docking on High-Performance Computing Machines”**

by LaBute et al.

**Figure S1. Protein structure quality control workflow used in preparation for docking calculations .**

**Table S1. ADR groupings and their MedDRA LLT side effect components .**

| **ADR GROUPS AND MEDRA LLTs** |
| --- |
| **neoplasms** |
| breast neoplasm |
| papilloma |
| adenocarcinoma |
| glioma |
| ovarian cyst |
| lung neoplasms malignant |
| basal cell carcinoma |
| **bloodAndLymph** |
| Idiopathic thrombocytopenic purpura |
| thrombotic thrombocytopenic purpura |
| agranulocytosis |
| **immuneSystem** |
| angioedema |
| sarcoidosis |
| vasculitis |
| **endocrineDisorders** |
| endocrine disorder |
| adrenal insufficiency |
| diabetic ketoacidosis |
| diabetic neuropathy |
| diabetic retinopathy |
| diabetes mellitus |
| dwarfism |
| hypopituitarism |
| diabetes insipidus |
| hyperparathyroidism |
| thyroiditis |
| thyroid disorder |
| hyperthyroidism |
| hypothyroidism |
| **psychDisorders** |
| panic disorder |
| delirium |
| psychomotor retardation |
| bipolar disorder |
| paranoia |
| borderline personality disorder |
| acute psychosis |
| schizophrenia |
| **cardiacDisorders** |
| aortic valve incompetence |
| atrial fibrillation |
| atrial septal defect |
| bundle branch block |
| bundle branch block right |
| cardiac arrest |
| cardiac tamponade |
| cardiomegaly |
| cardiomyopathy |
| cor pulmonale |
| endocarditis |
| myocardial infarction |
| myocarditis |
| pericarditis |
| rheumatic fever |
| sick sinus syndrome |
| sinus arrest |
| sinus tachycardia |
| supraventricular extrasystoles |
| supraventricular tachycardia |
| tachycardia |
| ventricular extrasystoles |
| ventricular fibrillation |
| ventricular septal defect |
| ventricular tachycardia |
| **vascularDisorders** |
| aneurysm |
| aortic aneurysm |
| arterial insufficiency |
| arteriosclerosis |
| venous insufficiency |
| venous thrombosis |
| deep vein thrombosis |
| thrombophlebitis |
| thrombophlebitis superficial |
| arteritis |
| **gastroDisorders** |
| pancreatitis |
| duodenal ulcer |
| gastric ulcer |
| intestinal ulcer |
| peptic ulcer |
| gastrointestinal haemorrhage |
| upper gastrointestinal haemorrhage |
| lower gastrointestinal haemorrhage |
| **hepatoDisorders** |
| cholangitis |
| hepatic encephalopathy |
| hepatic failure |
| liver abscess |
| hepatic necrosis |
| **renalDisorders** |
| nephrotic syndrome |
| nephropathy |
| renal failure |
